# Mr. Hume's Cursory Remarks on Arsenical Preparations

**Published:** 1810-11

**Authors:** Joseph Hume

**Affiliations:** Long Acre


### To the Editors of the Medical and Physical Journal.

### Mr. Hume's *Cursory Remarks on Arsenical Preparations.*

Gentlemen,

The internal application of arsenic to the human frame is of such moment in therapeutics, this drug having nothing in its character between the two extremes of doing a great deal of good, or much mischief, that any error respecting the preparations of an agent so formidable cannot be too soon or too generally exposed, particularly if such error be sanctioned by names of professional, and, in all other respects, most meritorious authors.

There are not more than two formulæ admitted into general practice in the British empire, viz. Fowler's solution, and Macquer's arsenical salt; the first of these may be considered as the *arsenite*,* and the other salt as the *arseniate* of potash, this alkali being the basis in both cases. In regard to another preparation, the simple solution of the white oxide in distilled water, first prescribed by M. Le Febure, it is, I believe, seldom employed in this country; but, considered as an officinal article, there is one objection against it, that it cannot be preserved long, being disposed to turn fetid, and of course to become impaired in its efficacy.

By Dr. Fowler's prescription, his solution ought to contain four grains of the oxide in each fluid ounce of the solution, or, which is equivalent, sixty-four grains in the English pint. The compound spirit of lavender may be useful to prevent mistakes, lest this mixture should pass for plain water, but as cheaper substances might be contrived, I apprehend the author's aim was to preserve this remedy from spoiling. This medicine is precisely the same form admitted by the London College into their pharmacopœia, but why it has been preferred to the arseniate I am not yet informed; it may be superior in its powers, or it may be equal to the arseniate of the Dublin College.

The mistake to which I now particularly allude is, that of confounding the two terms, *pound* and *pint;* for, unfortunately, the word *libra* has long been employed indiscriminate-

* As *i* is a radical letter in the primitive word, perhaps this should be written *arseniite*; I am not, however, disposed to contend this point, especially as the modern nomenclature is not only fleeting, but even degraded, through some of Mr. Davy's splendid discoveries.

ly for both, and probably we may trace the blunder to this source. This fault may be excusable in the Latin language, where there is less choice, but as it really prevails among some English authors, I trust this observation will have its weight and be considered equally as interesting to patients as it is to physicians. Now this error, the unqualified use of the words pint and pound, in one example at least, appears in the same pages of the same volume; therefore, should Fowler's solution be prepared from such an authority, is there not a chance of twelve ounces troy *weight* being substituted for the pint, or, what is now deservedly preferred, the octarius of the College here?—The technical excellence of the work itself and the high character of the author, to whom I more closely refer, leave no room to doubt that this ambiguity will be removed in a future edition.

In regard to the discordance which exists in our metropolitan pharmacopœias, there is, notwithstanding some late improvements, still great room for complaint. This important subject was, I have reason to believe, first taken up by myself, by those general observations which are inserted in the 11th and 12th vol. of your Journal, and which must be considered as a mere attempt to encourage others to pursue the same route, who have more leisure and perhaps more knowledge upon this important matter. I cannot subscribe to the necessity of invoking the royal authority to sanction three pharmacopœias for the united kingdom, while these volumes, speaking of them collectively, teem with dissimilarity in names, compounds, materia medica, and indeed in all other particulars. Surely the constitutions of the English, Irish, and Scots do not require each a distinct set of medicines or a peculiar mode of treatment in any disease whatever; why, therefore, such discongruity in these prescriptions?

This reminds me of my original subject, the arsenical preparations. No reason, I conceive, has yet been assigned why the Dublin College adopts the arseniate of potash, while the London College prefers Fowler's arsenite. If these medicines possess the same powers in all respects, it is unnecessary for the dispensatories still to continue thus diverging; I should think some scheme might be formed to ascertain the respective merits of these two articles at least. Should they prove to be of equal value, I would not hesitate to adopt the arseniate, or Macquer's neutral salt, for there is not a more neat and perfect chemical compound; it is portable, durable, and in every respect most suitable for officinal preference.

The process of the Dublin College for this salt might in some measure be improved, but here I speak with great deference; the heat I consider as too great, for, if the bottom of the glass vessel be even obscurely red, some part of the arseniate will be decompounded; I would, therefore, rather say, "igne sensim aucto donec cessaverint vapores, &c."

We know, and it is generally admitted, that a wide difference exists between some mercurial compounds, between calomel and corrosive sublimate as an example; that these produce distinct effects on the human frame, and that practitioners to this day are not quite agreed as to their precise value. I mention this circumstance as it serves to illustrate the following comparison, in which, as far as chemical tests can decide, there is the same opposition in the two preparations of arsenic.

Thus, lime water changes calomel into a *black* oxide or precipitate, and corrosive sublimate into a bright *yellow* oxide of mercury, colors certainly perfectly opposite to each other; and in respect to the arsenical compounds, I have, in my late communications to you, fully described the effect of nitrate of silver, which converts the metal of the arsenite into a bright *yellow* precipitate, being arsenite of silver; while the same reagent turns the arseniate into a deep brick-coloured *red*. Whether these habitudes of the arsenical salts prognosticate an important variety in them, as medicines, is a question yet to be determined.

It is chiefly in public hospitals, both naval and military, as well as in charitable institutions, that true results can be obtained to guide such a decision, and the relative merits of these and many other remedies fairly appreciated; to the managers of these numerous and proud marks of British charity, let us therefore with confidence assign the investigation of this and all similar doubts.

I beg now to testify my obligations to your correspondent, Mr. Jones, for his ready attention to my request, and to say that, having no reason to doubt the accuracy of his conducting the experiments which I suggested, I fully acquiesce in the deductions he has drawn respecting the case of poison by arsenic.

As, in the process of heating the suspected arsenic with nitrate of potash, I rather insist more upon the *effect* than the *color* of the nitrous vapors or acid that might arise, let me enforce the necessity of watching the *change* produced on the litmus-paper; for if the blue become reddish, one proof at least of the presence of the poison is substantiated. In case litmus paper be not at hand we may substitute many other vegetables, such as a strong infusion of red-rose flowers, mallow-flowers, red-cabbage, &c.; by dipping a piece of any clean white paper that does not readily bear ink into one or other of the above infusions.

I remain, Gentlemen, Your obedient servant,

*Long Acre, Oct.* 13, 1810. JOSEPH HUME.

